# Supplementary material for: Dried Blood Spots (DBS): A suitable alternative to using whole blood samples for diagnostic testing of visceral leishmaniasis in the post-elimination era
Source: PLoS Negl Trop Dis. 2023 Oct 20;17(10):e0011680. doi: 10.1371/journal.pntd.0011680 (PMC10588855; doi:10.1371/journal.pntd.0011680)
Supplement: S1 Table — (DOCX) [file pntd.0011680.s001.docx]

Table: The primers and probe sequences of real time PCR

| **Primers & Probe** | **Sequences** | **Neucleotide position** |
| --- | --- | --- |
| Forward Primer | 5’- GCGACGTCCGTGGAAAGAA-3’ | 77–95 |
| Reverse Primer | 5‘-GGCGGGTACACATTAGCAGAA-3’ | 122–142 |
| Probe | 5’ -CAACGCGTATTCCC- 3’ | 108–121 |
