# Supplementary material for: Dried Blood Spots (DBS): A suitable alternative to using whole blood samples for diagnostic testing of visceral leishmaniasis in the post-elimination era
Source: PLoS Negl Trop Dis. 2023 Oct 20;17(10):e0011680. doi: 10.1371/journal.pntd.0011680 (PMC10588855; doi:10.1371/journal.pntd.0011680)
Supplement: S2 Table — (DOCX) [file pntd.0011680.s002.docx]

Table: The primers and probe sequences of RPA assay

| **Primers & Probe** | **Sequence (5’-3’)** | **Amplicon length** |
| --- | --- | --- |
| Forward Primer | ATGGGCCAAAAACCCAAACTTTTCTGGTCCTC | 160 bp |
| Reverse Primer | CTCCACCCGACCCTATTTTACACCAACCCCCAGT |  |
| Probe | CGCCTCGGAGCCGAT(BHQ1dT)(Tetrahydrofuran)(FAMdT) TGGCATTTTTGGCTATTTTTTGAACGGGAT-phosphate |  |
