# Supplementary material for: Dried Blood Spots (DBS): A suitable alternative to using whole blood samples for diagnostic testing of visceral leishmaniasis in the post-elimination era
Source: PLoS Negl Trop Dis. 2023 Oct 20;17(10):e0011680. doi: 10.1371/journal.pntd.0011680 (PMC10588855; doi:10.1371/journal.pntd.0011680)
Supplement: Attachment — Submitted filename: Review Report.docx [file pntd.0011680.s003.docx]

Review Report:

The manuscript “Dried Blood Spots (DBS): a suitable alternative to using whole blood samples for diagnostic testing of visceral leishmaniasis in the post-elimination era” has compared dried blood samples in comparison to whole blood NA for qPCR and RPA and serum for ELISA. Dried blood samples are being used to diagnose Leishmaniasis for years. In many areas, it was the sample of choice to diagnose Leishmaniasis because of comfortable transportation and storage. It was used mostly for sero-diagnosis (DAT and ELISA) and various molecular diagnostics. But now in the presence of rapid diagnostic kit; importance of DBS is not as much because the former needs only one blood drop from finger puncture and result comes within few minutes and don’t need much expertise and lab wares. Hence importance of this method is not as much in post RDK/RDT. However, it is a good effort to keep an alternative ready for the situations like non availability of RDK/RDT on the spot. Since the data shows compromised result in qPCR and RPA, the DBS cannot be an alternative of fresh sample. The proposed system is also as invasive as fresh blood collection. Fig. 1 and 2 is not visible. It must be uploaded rechecked before its final submission. In addition, following points should be addressed:

1. Which Whatman FTA card was used? There are various categories.
2. What was the basis of using two 5mm circles to elute DBS in 150 and 200μl elution buffer? Further, how the authors achieved 1:2000 dilutions from DBS? Previously Harith et al. 1989, Bimal et al. 2005 and many others had eluted single 5mm circle in 165 μl elution buffer to get 1:50 dilution of antibodies. Quantification may be explained in the text.
3. After how long the samples collected were eluted? It also may be indicated in figure 7.
4. The sample was stored at -20^o^C till further use. How long it was stored? Prolonged storage in -20^o^C loose Ab titer and protein.
5. Statistical significance may be rechecked by an statistician for Fig. 3
6. Primers used may be provided in the text for consistency?
7. Invasive statement may be changed/ corrected
8. Fig. 1 and 2 must be uploaded with good resolution.
