# Supplementary material for: Dried Blood Spots (DBS): A suitable alternative to using whole blood samples for diagnostic testing of visceral leishmaniasis in the post-elimination era
Source: PLoS Negl Trop Dis. 2023 Oct 20;17(10):e0011680. doi: 10.1371/journal.pntd.0011680 (PMC10588855; doi:10.1371/journal.pntd.0011680)
Supplement: Attachment — Submitted filename: Letter with review responses_PNTD.docx [file pntd.0011680.s004.docx]

Dear Editor

PLOS Neglected Tropical Diseases

We would like to submit the review responses on the manuscript titled **“Dried Blood Spots (DBS): a suitable alternative to using whole blood samples for diagnostic testing of visceral leishmaniasis in the post-elimination era”** by Prakash Ghosh et al for your kind consideration. Please see the below responses against each of the comments.

**Methods**

**Reviewer #1:** The hypothesis that continued surveillance must be done to preserve the gains made by the elimination campaign is widely accepted in the VL research community. However, the insistence that serology will be a good indicator of any emerging foci needs to be clarified. Anti-rK-39 and rK-28 antibodies are found at high levels in South Asian endemic regions, compared to East Africa and are long lasting. Hence sero-surveys though useful in assessing endemicity, may not consistent predictors of new infections, which is the key need post- elimination. PCR may be a better predictor of that. These points need to be considered in the rewriting of the draft.

***Response:*** *Thanks to the reviewer for thoughtful comments.* *Indeed, the application of Anti-rK-39 antibody to identify VL infection is not appropriate for both the old and new world. In the Indian subcontinent the elimination programs have been successful in reducing the number of VL cases. Particularly in Bangladesh the program has achieved the elimination goal. At peri and post elimination period the infection rate will be so low eventually the waning level of antibodies against leishmanial antigens(rK-39&rK-28) is expected. Thus, the chances of the detection of parasite DNA in blood will be low. To date, the longitudinal studies towards establishing serological markers as the predictors of VL infection is scarce. However, a recent study by Mondal et al.* *showed that a certain titer of rK-39 antibody can predict asymptomatic infection progression to VL within 24 months by 87.5% sensitivity and 95% specificity^1^. The findings are in favor of implementing the serological biomarkers as a surveillance tool to predict VL infection. However, molecular methods including conventional PCR, qPCR and RPA assay can be done to detect parasite DNA for confirmation.*

**Reviewer #2:** Methodology part need some improvements as suggested. The statement of invasive needs to change with appropriate word as DBS collection is as invasive as fresh sample collection i.e. use of needle.

***Response:*** *The methodology section has been revised as per reviewer’s suggestion.*

**Results:**

**Reviewer #1:** The results and analyses and their representations are sufficient for the serological tests. Statistical methods are appropriate. However, for the PCR methods, more details must be included in the results section for clarity and completion.

***Response:*** *The* *findings of molecular assays (qPCR & RPA) have already been provided in the result section. Moreover, a detailed table (table-2) portraying the findings of the molecular assays has been added.*

**Reviewer #2:** Result is OK. P value of fig. 3 may be reevaluated.

***Response:*** *Figure 3 has been revised as per reviewer’s suggestion.*

**Conclusions**

**Reviewer #1:** The conclusions are in line with the results mostly. However, the limitations of the analysis must be more clearly discussed. The sample size is mentioned, and the inclusion of a larger sample size is alluded to. This is to be expected. But, given the success of the elimination campaign, it is highly likely that larger a larger cohort of VL patients is not available. The authors must mention this and how it will impact any future studies.

Apart from that, the authors must also clearly state the advantages and disadvantages of using serology form surveillance in endemic foci. This has been a subject of extensive discussion in the VL field and how other techniques can fill the gap, namely PCR and antigen detection urine tests. That is a clear limitation of the study and DBS utility, which has not been discussed in this manuscript.

***Response:*** *The authors have adequately described the limitation of the study in discussion. Considering the small sample size of the current study the authors have suggested large scale cohort study to discern the application of the investigative methods. Since the reviewer has pointed out that small number of cases are expected in post elimination period, therefore, multicounty study would overcome the challenges owing to small sample size.*

*In the last decade, numerous studies have been conducted in several settings to explore the effectiveness of serology-based methods for both diagnosis and surveillance of VL. Despite the limitations of serology methods in differentiating between present and past infection, the serology-based methods, specially rK-39 RDT has played crucial role in the Indian subcontinent towards eliminating the VL. To date, several molecular tests have been developed for diagnosis of VL. However, application of this method for surveillance is very limited as scanty of parasite are found in the peripheral blood at asymptomatic stage. In addition, several urinary based tests have been developed for diagnosis of VL. However, large scale clinical studies are still needed to be implemented as surveillance tool in the endemic settings. Here to note, in the chronic VL patients the kidney function is impaired, therefore the chance of excretion of leishmania specific protein particles is very high, whereas the chances is very low in asymptomatic individuals.*

**Reviewer #2:** Conclusion is well discussed but the importance of this study is not as much important as claimed. Need to address the point

***Response:*** *The authors have tried to explore the promises of DBS followed by rK-39 and rK-28 ELISA assays as a means of surveillance at post elimination of VL. Considering the sample size, it is too early to completely comprehend the applicability of the findings of the study. However, the study has provided some rudimentary findings which will guide future large-scale clinical studies to establish a cost effective and feasible DBS based surveillance strategy for post elimination period of VL in the endemic settings.*

**Editorial and Data Presentation Modifications**

**Reviewer #1:** The authors must include the discussion on the limitations of sero-surveillance that are suggested above in the revised manuscript.

The authors must include the Limit of detection of both the qPCR and RPA-PCR for whole blood and DBS in the results section. They only disclose the Ct and TT values, not the absolute levels of parasite genome equivalents or DNA concentrations that are detected by the methods. It is also necessary that the authors disclose the yield of DNA from DBS, and discuss its correlation to whole blood, since it is a highly likely contributor to the differences in sensitivity.

These details must be included in the revised manuscript.

***Response:*** *We measured Limit of detection of qPCR assay in detecting Leishmania donovani parasite in our previous study which is 10 fg of Leishmania donovani genomic DNA per reaction, a quantity that corresponds to 0.1 parasite^2^ and the LD RPA assay detected down to one LD genomic DNA^3^.* *So, we did not measure the limit of detection in this study.*

*In the current study 200 uL peripheral blood was used to extract DNA. On the other hand, three punches of DBS containing 24 uL blood was used to extract DNA. Eventually the DNA yield from whole blood will be higher than that of DBS.*

**Reviewer #2:**

1. Which Whatman FTA card was used? There are various categories.

***Response:*** *Whatman FTA cards from Sigma-Aldrich was used. We have used the same filter paper in our previous study^4^.*

2. What was the basis of using two 5mm circles to elute DBS in 150 and 200 micro lit. elution buffer? Further, how the authors achieved 1:2000 dilutions from DBS? Previously Harith et al. 1989, Bimal et al. 2005 and many others had eluted single 5mm circle in 165 micro lit. elution buffer to get 1:50 dilution of antibodies. Quantification may be explained in the text.

***Response:*** *The dilution in the previous version of the manuscript was with a Typo error. We obtained 1:200 dilutions where 1.5 uL FP sample were diluted in 298.5 uL diluent.*

3. After how long the samples collected were eluted? It also may be indicated in figure 7.

***Response:*** *Elution was done after 2.5 years from the impregnation of blood. Figure 7 is a proposal of a surveillance strategy from the authors. It is not part of the study.*

4. The sample was stored at -20°C till further use. How long it was stored? Prolonged storage in -20°C loose Ab titer and protein. Definitely there is a value of deterioration. So, actual storage time must be recorded in methodology.

***Response:*** *DBS was stored in -80° C for 2.5 years. According to the previous study performed by et al, DBS samples can be stored for at least 3 years in the mentioned condition without impacting  the Ab titer^5^. The extracted DNA was stored in -20 C for a couple of months prior downstream analysis. ELISA was performed in the following day of elution of DBS.*

5. Statistical significance may be rechecked by an statistician for Fig. 3

***Response*:** *Revised figure has been added to the manuscript according to the reviewer’s suggestion.*

6. Primers used may be provided in the text for consistency?

***Response:*** *The primer and probe sequences have been incorporated in the supplementary data 1- table-01 & table-02.*

7. Invasive statement may be changed/ corrected. Isolation of DBS is not non-invasive.

***Response:*** *The authors wanted to convey that the collection of finger prick blood is a minimally invasive procedure which is followed by impregnation of blood sample in filter paper.*

8. Fig. 1 and 2 must be reuploaded with good resolution and background.

***Response:*** *Revised figures have been included in the manuscripts as per reviewer’s suggestion****.***

**Summary and General Comments**

**Reviewer 1:** DBS is very promising for sero-surveillance and has been successfully implemented for other NTDSs such as filariasis that are also part of elimination campaigns. This is the first instance of a systematic study of DBS utility in the VL elimination campaign of South Asia.  The authors have used the study to state the utility of DBS in Bangladesh clearly and precisely. The general execution, ethics, statistics and writing are very good and the authors clearly state the hypotheses and results.  With the above suggested minor revisions, the manuscript is suitable for publication.

***Response:*** *We would like to thank the reviewer for expressing his/her positive opinion and suggestions to amend the manuscript and subsequent publication.*

**Reviewer 2:** The manuscript “Dried Blood Spots (DBS): a suitable alternative to using whole blood samples for diagnostic testing of visceral leishmaniasis in the post-elimination era” has compared dried blood samples in comparison to whole blood NA for qPCR and RPA and serum for ELISA. Dried blood samples are being used to diagnose Leishmaniasis for years. In many areas, it was the sample of choice to diagnose Leishmaniasis because of comfortable transportation and storage. It was used mostly for sero-diagnosis (DAT and ELISA) and various molecular diagnostics.   But now in the presence of rapid diagnostic kit; importance of DBS is not as much because the former needs only one drop blood from finger puncture and result comes within few minutes and don’t need much expertise and lab wares.  Hence importance of this method is not as much in post RDK/RDT. However, it is a good effort to keep an alternative ready for the situations like non-availability of RDK/RDT on the spot.

Since the data shows compromised result in qPCR and RPA, the DBS cannot be an alternative of fresh blood sample. The proposed system is also as invasive as fresh blood collection.

***Response:*** *Thank you for reviewing the manuscript and putting valuable comments which are critical to rectify the manuscripts. We agree with the reviewer that the rK-39 RDT is more convenient to perform at the field level for surveillance and epidemiological studies as well. At peri and post elimination period the infection rate will be so low eventually the waning level of antibodies against leishmanial antigens(rK-39&rK-28) is expected. A recent study by Mondal et al. showed that a certain titer of rK-39 antibody can predict asymptomatic infection progression to VL within 24 months by 87.5% sensitivity and 95% specificity. Therefore, it is evident that the rK-39 & rK-28 ELISA is not only an alternative tool to RDK/RDT for surveillance but also has* *the potential to predict the VL infection which would have paramount impact in achieving the zero-transmission goal at post elimination period of VL.*

**References:**

1.	Mondal D, Ghosh P, Chowdhury R, et al. Relationship of Serum Antileishmanial Antibody With Development of Visceral Leishmaniasis, Post-kala-azar Dermal Leishmaniasis and Visceral Leishmaniasis Relapse. *Front Microbiol*. 2019;10(October):1-12. doi:10.3389/fmicb.2019.02268

2.	Hossain F, Ghosh P, Khan MAA, et al. Real-time PCR in detection and quantitation of Leishmania donovani for the diagnosis of Visceral Leishmaniasis patients and the monitoring of their response to treatment. *PLoS One*. 2017;12(9):e0185606. doi:10.1371/journal.pone.0185606

3.	Mondal D, Ghosh P, Khan MAA, et al. Mobile suitcase laboratory for rapid detection of Leishmania donovani using recombinase polymerase amplification assay. *Parasites and Vectors*. 2016;9(1). doi:10.1186/s13071-016-1572-8

4.	Hossain F, Picado A, Owen SI, et al. Evaluation of Loopamp^TM^ Leishmania Detection Kit and Leishmania Antigen ELISA for Post-Elimination Detection and Management of Visceral Leishmaniasis in Bangladesh. *Front Cell Infect Microbiol*. 2021;11(April). doi:10.3389/fcimb.2021.670759

5.	Bevins S, Pappert R, Young J, Schmit B, Kohler D, Baeten L. Effect of storage time and storage conditions on antibody detection in blood samples collected on filter paper. *J Wildl Dis*. 2016;52(3):478-483. doi:10.7589/2015-09-242

Thank you for your time.

Regards,

Dinesh Mondal, MD, PhD

Senior scientist, icddr,b

Corresponding author of this article

Mail address: din63d@icddrb.org

**Corresponding Authors:**

Dinesh Mondal, MD, PhD

Senior scientist, icddr,b

Mail address: din63d@icddrb.org

Phone: +88-01712027091

&

Prakash Ghosh, MSc

Assistant Scientist, icddr,b

Mail address: dreamix888@gmail.com

Phone: +88-01731855586
